# CO_2_ Gasification Reactivity of Char from High-Ash Biomass

**DOI:** 10.1021/acsomega.1c05728

**Published:** 2021-11-29

**Authors:** Aekjuthon Phounglamcheik, Ricardo Vila, Norbert Kienzl, Liang Wang, Ali Hedayati, Markus Broström, Kerstin Ramser, Klas Engvall, Øyvind Skreiberg, Ryan Robinson, Kentaro Umeki

**Affiliations:** † Division of Energy Science, Luleå University of Technology, SE-971 87 Luleå, Sweden; ‡ Material Science and Environmental Engineering, Tampere University, FI-33720 Tampere, Finland; § BEST—Bioenergy and Sustainable Technologies GmbH, Inffeldgasse 21b, 8010 Graz, Austria; ∥ SINTEF Energy Research, P.O. Box 4761 Torgarden, 7465 Trondheim, Norway; ⊥ Department of Applied Physics and Electronics, Thermochemical Energy Conversion Laboratory, Umeå University, SE-901 87 Umeå, Sweden; # Division of Fluid and Experimental Mechanics, Luleå University of Technology, SE-971 87 Luleå, Sweden; ¶ Department of Chemical Engineering, KTH Royal Institute of Technology, SE-100 44 Stockholm, Sweden; ∇ Global Technology, Höganäs AB, SE-263 83 Höganäs, Sweden

## Abstract

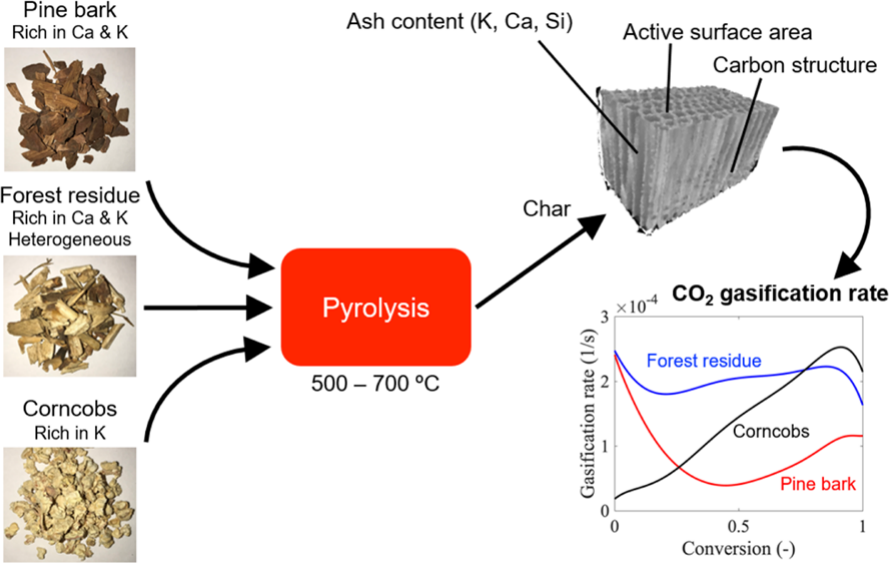

Biomass char produced from pyrolysis processes is of great interest to be utilized as renewable solid fuels or materials. Forest byproducts and agricultural wastes are low-cost and sustainable biomass feedstocks. These biomasses generally contain high amounts of ash-forming elements, generally leading to high char reactivity. This study elaborates in detail how chemical and physical properties affect CO_2_ gasification rates of high-ash biomass char, and it also targets the interactions between these properties. Char produced from pine bark, forest residue, and corncobs (particle size 4–30 mm) were included, and all contained different relative compositions of ash-forming elements. Acid leaching was applied to further investigate the influence of inorganic elements in these biomasses. The char properties relevant to the gasification rate were analyzed, that is, elemental composition, specific surface area, and carbon structure. Gasification rates were measured at an isothermal condition of 800 °C with 20% (vol.) of CO_2_ in N_2_. The results showed that the inorganic content, particularly K, had a stronger effect on gasification reactivity than specific surface area and aromatic cluster size of the char. At the gasification condition utilized in this study, K could volatilize and mobilize through the char surface, resulting in high gasification reactivity. Meanwhile, the mobilization of Ca did not occur at the low temperature applied, thus resulting in its low catalytic effect. This implies that the dispersion of these inorganic elements through char particles is an important reason behind their catalytic activity. Upon leaching by diluted acetic acid, the K content of these biomasses substantially decreased, while most of the Ca remained in the biomasses. With a low K content in leached biomass char, char reactivity was determined by the active carbon surface area.

## 1 Introduction

Biomass char is a promising renewable solid fuel that has the potential to convert the fossil-based industry toward net-zero emission. To achieve climate neutrality by 2050,^1−3^ the industrial sector has been required to carry out immediate development and implementation of renewable energy and feedstocks. The steel industry contributes around 20% of GHG (greenhouse gas) emissions from the industrial sector in the EU.^4^ Suopajärvi et al.^5^ summarized the state of the art to utilize biomass char as alternative reductants in various steelmaking processes. It has been reported that biomass char can fully replace pulverized coal injection^6^ and partially replace coke in blast furnaces.^7^ Biomass char can also replace carburization media in electric arc furnaces.^8^ However, the steel industry still has not implemented biomass char in commercial processes although the literature clearly states the potential for it as a substitute for fossil coal and coke.

Previous studies showed that the elemental composition and heating value of woody biomass char can be close to fossil coal when producing it through pyrolysis at a temperature higher than ≥500 °C.^9,10^ However, the mass yields of char decreased from ca. 38–18% when the pyrolysis temperature increased from 300 to 700 °C.^10,11^ Nevertheless, a debarked woodchip, that is, stem wood without bark, is considered less sustainable and expensive because of its highly competitive demands on the industrial market. On the other hand, forestry byproducts, such as branches, residues, and bark, as well as agricultural wastes, such as corncobs, straw, and rice husk, have relatively low prices. Therefore, these alternative resources should be considered to increase the economic feasibility of biomass char production.

The ash content and relative composition of ash-forming elements vary significantly among different types of biomasses. Forestry byproducts and agricultural wastes generally contain a much higher amount of inorganic elements than stem wood.^12−15^ For instance, Werkelin et al.^16^ reported that bark has around ten times higher total inorganic content than stem wood, particularly Ca. Forest residues are known to contain heterogeneous compositions of Ca, K, and Si.^13^ In addition, agricultural wastes such as corncobs contain a very high K content.^13,14,17^ These elements have strong impacts on ash formation and ash-related operational problems during thermochemical conversion processes, for example, for the implementation of biomass char in steel production. The presence and transformation of these elements affect conversion behavior and char reactivity,^18,19^ thus interfering with the steel making process and the quality of the final products.

The intrinsic gasification rate of biomass char is relatively high compared to fossil coal. It has been reported that biomass char reacts four times faster than anthracite coal in gasification at 850–1000 °C.^20^ This is another limitation to utilize biomass char as a reducing agent in the iron reduction processes. Thus, the intrinsic reactivity of char was studied,^21−24^ and the three main parameters influencing char reactivity were given: (i) the pore-size distribution and specific surface area, (ii) carbon structure and the content of functional groups, and (iii) the content, composition, and chemical speciation of inorganic elements. The internal surface area and carbon structure of biomass char represent a fraction of the active sites available on the accessible char surface. As reported in our previous study,^25^ these properties are strongly influenced by pyrolysis conditions, particularly the temperature and heating rate. It is also important to mention that the effects of some pyrolysis parameters can show an opposite trend when char is produced from biomass powder or large particles. Large particles are more likely to be used for biomass char production at an industrial scale to avoid high particle grinding energy and to gain higher char yield.^26^ However, most studies use powder samples to investigate the effects of pyrolysis conditions on gasification reactivity, which may give different outcomes compared to large particles. Therefore, it is necessary to examine the correlation between pyrolysis conditions and the gasification reactivity of char produced from large samples.

Alkali and alkaline earth metals (AAEM), for example, Na, K, Ca, and Mg, tend to form carbonates during char gasification, which enhances the reaction rate due to their catalytic effect via the oxygen transfer cycle.^27−31^ In the meantime, Cl, S, P, and Si have a possibility to react with AAEM, which reduces the availability of the catalytic compounds.^32,33^ The content and distribution of these inorganic elements in char depend strongly on the type of biomass.^16^ K, Ca, Mg, Si, P, S, Cl, and Al are generally considered to be the major ash-forming elements in a biomass.^33^ Biomass char with a low content of Cl, S, P, and Si has a high potential to form Ca-carbonates and K–Ca-carbonates during thermochemical conversion processes.^33^ Nevertheless, the stability of these carbonates is highly dependent on the Ca-to-K ratio and reaction atmosphere.^30^ Strandberg et al.^30^ reported a thermodynamic equilibrium calculation during the combustion of stem wood. The result shows that under certain conditions, K is released from the solid phase at a temperature above 800 °C. The release of K results in the mobility of K during the conversion of biomass particles, possibly leading to higher catalytic enhancement. Schneider et al.^34^ found that a thin layer of CaO was formed in the biomass char produced at 1600 °C, which was not present in char produced at lower temperatures, resulting in a significant increase in the gasification rate. These results imply that the catalytic effect caused by K and Ca vary greatly with pyrolysis and gasification conditions. Moreover, the content of K- and Ca-carbonates is lower in biomass char with higher concentrations of Cl, S, P, and Si. Boström et al.^33^ reported that Si tends to form K-silicates followed by Ca-silicates. Consequently, only individual contents of K and Ca are not sufficient to predict the gasification reactivity of biomass char.

Most of the Cl and S in biomass is usually released during the pyrolysis process together with some K, while Ca, Si, and P remain in the char.^5,35,36^ According to the literature, K starts to release at temperatures above 700 °C, but the release of Ca and Si is negligible below 900 °C.^37−41^ Therefore, char produced from these biomasses usually contain high amounts of Ca and Si, and some amount of K. These inorganic elements in a biomass can be removed by solvent extraction. Water washing could remove water-soluble elements, for example, the majority of K and Cl, but it has minor effects on the concentrations of some elements such as Ca, Si, and Mg.^42^ Acid leaching using a strong acid effectively removes these inorganic elements.^41,43−45^ Anca-Couce et al.^41^ leached pine wood using 37% HCl solution and achieved >90% decreases in total ash, Ca, and K contents. Using strong acids give better leaching performance but reduce the volatile matters in biomass due to the dissolution of hemicellulose.^43,44^ In addition, inorganic acids such as HCl, HNO_3_, and H_2_SO_4_ could add impurity, such as Cl and S, in char. Hence, organic acids, such as acetic acid, are alternatives to avoid problematic issues. As reported by Persson et al.,^45^ leaching of spruce/pine sawdust by 10% (mass basis) acetic acid can decrease the ash content from 3.5 to <1%, while volatile matters remain constant. The result also showed significant decreases in Ca and K in the leached biomass. Nevertheless, the performance of acid leaching may differ among different types of biomasses. Particularly, large particle sizes may hinder the leaching performances compared with biomass powder. Therefore, acid leaching in thick high-ash biomasses may result in different inorganic contents in the produced char, resulting in different gasification reactivities. To the best of our knowledge, this is not well elaborated previously.

This study aims to elaborate on how to control gasification reactivity of char produced from large particles of high-ash biomass. The objective is to investigate effects of chemical and physical properties on CO_2_ gasification rates of high-ash biomass char and to understand the interactions between these properties. Acid leaching was considered as pretreatment and its performance in large biomass particles was examined. Pine bark, forest residue, and corncobs are raw biomasses used in this study. Biomass char was produced using a macro-thermogravimetric (macro-TG) reactor at different pyrolysis temperatures. Characterization of the char samples included elemental composition, morphology, and aromatic carbon structure. The gasification reactivity of ground char samples was measured using thermogravimetric analysis (TGA) under an isothermal condition.

## 2 Materials and Methods

### 2.1 Sample Preparation

Biomass chars used in this study were prepared from three different types of biomasses, including pine bark, pine forest residue, and corncob. The selection of these materials is based on their availability in Sweden, which are also the main forest byproducts and agricultural wastes in many countries. Furthermore, the selected biomasses have a high content of relevant inorganic elements influencing gasification rates, that is, Ca, K, and Si. Table 1 shows the lignocellulosic composition of the raw biomasses measured according to the TAPPI standard (T222 and T249) and FCBA method.

**Table 1:** Lignocellulosic Composition of Raw Biomasses (% on Dry Mass Basis)

properties	pine bark	pine forest residue	corncob
cellulose	21.9	22.3	38.4
hemicellulose	18.3	27.9	34.8
lignin	40.7	27.6	15.9
extractives	15.2	18.9	6.9

The representative samples of raw biomass were prepared by using the coning and quartering method according to ISO 14780:2017. The biomass was screened using stainless steel sieves with the mesh aperture width between 4 and 30 mm. The selected material was dried in an oven at 105 °C for 24 h. Dried biomasses were divided into smaller portions by using a rotary divider, Retsch PT100, to obtain smaller batches of representative biomasses.

In this study, some of the prepared biomasses underwent acid leaching to remove most of the inorganic compounds. Around 100 g of biomass was soaked in 1 L of 10% (mass basis) acetic acid in a closed volumetric flask. The sample was stirred by using magnetic stirrers at the rotation speed of 400 min^–1^, while the temperature was kept at 80 °C. After 24 h of the soaking step, the biomass was rinsed with deionized water until the pH of rinsed water became neutral. Then, the leached biomass was dried in an oven at 105 °C for 24 h. Afterward, the leached biomass was divided into smaller portions by using a rotary divider, Retsch PT100. Table 2 summarizes the proximate analysis, ultimate analysis, and major ash-forming elemental composition of original biomasses and leached biomasses. The measurement methods are described in a later section. It should be noted that the oxygen content was measured directly in the samples, thus the summation of elemental composition did not reach 100%.

**Table 2:** Proximate and Ultimate Analysis of Original Biomasses and Leached Biomasses

	pine bark	forest residue	corncob	leached pine bark	leached forest residue	leached corncob
Proximate Analysis (% Dry Mass Basis)						
fixed carbon	17.1	12.8	13.8	22.2	14.3	11.1
volatile matter	78.7 (±0.3)	85.0 (±0.5)	82.2 (±0.1)	77.1 (±0.6)	85.6 (±0.5)	88.6 (±0.2)
ash content	4.2 (±0.4)	2.2 (±0.4)	4.0 (±0.6)	0.7 (±0.1)	0.1 (±0.1)	0.3 (±0.0)
Ultimate Analysis (% Dry Mass Basis)						
C	50.6 (±0.4)	50.7 (±0.1)	47.2 (±0.2)	54.2 (±0.1)	51.7 (±0.4)	48.1 (±1.0)
H	6.1 (±0.2)	6.1 (±0.0)	5.9 (±0.0)	5.9 (±0.0)	6.2 (±0.0)	6.0 (±0.0)
N	0.5 (±0.0)	0.3 (±0.0)	0.5 (±0.1)	0.3 (±0.0)	0.3 (±0.0)	0.4 (±0.0)
O	40.5 (±1.9)	39.8 (±0.3)	42.8 (±0.3)	36.6 (±0.4)	39.8 (±0.1)	43.8 (±0.1)
Major Ash-Forming Elements (mg kg^–1^, Dry Basis)						
Al	537	76	<20	216	21	<12
Ca	4650	1590	128	2440	498	182
Fe	80	39	18	30	19	21
K	1840	1130	6310	188	103	418
Mg	582	327	273	125	72	65
Mn	238	164	3.7	51	28	1.1
Na	27	35	15	72	34	44
P	384	177	242	124	37	62
S	242	141	214	187	80	155
Si	480	515	435	99	180	215
Ti	4.1	2.0	<1	<2	<2	<1
Zn	35	25	13	16	<7	5

**Figure 1:**
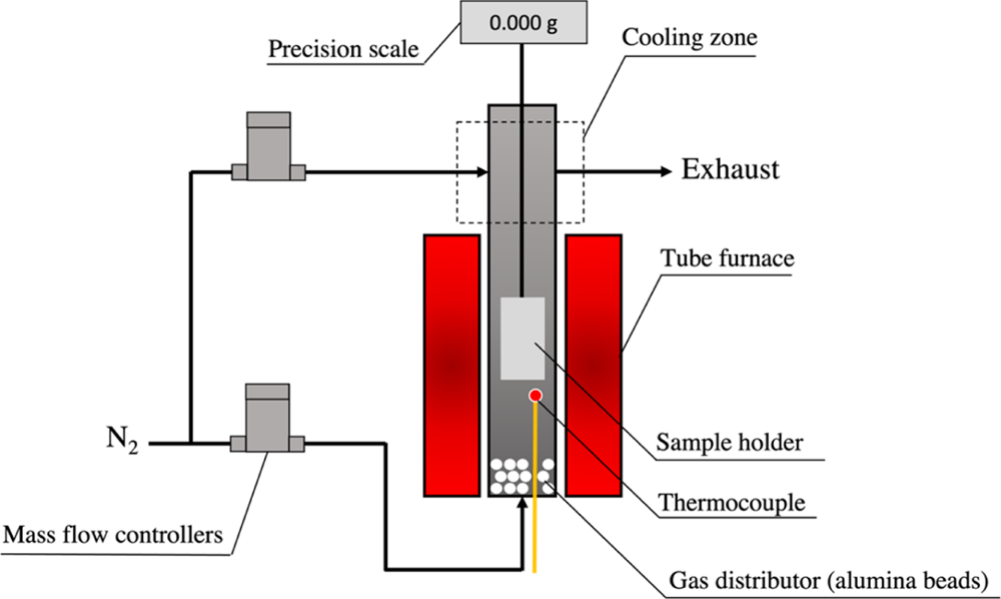
Macro-TG reactor.

After the preparation, original biomasses and leached biomasses were available to produce char samples. Char samples were produced by using a macro-TG reactor. The reactor is an externally heated stainless steel cylinder (grade 253 MA) with an internal diameter of 100 mm and a 450 mm long heating zone. A wire mesh basket (30 × 30 × 45 mm) was used to hold the sample. The sample holder was suspended to a precision balance from the top of the reactor chamber. The reactor temperature was measured by a type N thermocouple placed at the center of the reactor and 20 mm below the sample holder. The carrier gas enters the reactor from the bottom and leaves at the top of the reactor together with volatile gases generated during the experiment. Figure 1 shows a schematic drawing of the macro-TG reactor.

Prior to the char preparation, the macro-TG reactor was preheated to the reaction temperatures, that is, 500, 600, and 700 °C. The carrier gas was N_2_ (purity ≥ 99.996%) with a total flow rate of 7 L min^–1^ at the standard state. Around 5–10 g of biomass was filled into the wire mesh basket and was lowered manually down to the heating zone, typically within 2–3 s. The sample was held in the reactor for 5 min to complete major biomass devolatilization, while longer residence time may cause thermal annealing. Mass and temperature during the experiment were continuously recorded. Then, the sample was relocated to the cooling zone, where the sample was cooled down under an N_2_ atmosphere and kept for 5 min before taking out to the room atmosphere. The char samples were ground in a mortar and sieved to a sieve size below 75 μm. All experimental conditions had five repetitions to cover the deviation caused by the heterogeneous nature of the feedstocks. Table 3 shows the sample labels for each condition. The letters B, R, and C represents raw biomasses, that is, pine bark, forest residue, and corncobs, respectively, while the following number refers to pyrolysis temperatures. The char samples prepared from leached biomasses have the additional letter “L” at the beginning of the labels. For instance, “LB500” means char prepared from leached pine bark at pyrolysis temperature of 500 °C.

**Table 3:** Pyrolysis Conditions and Labels of Char Samples

sample label	type of biomass	leaching	pyrolysis temperature (°C)
B500	pine bark	none	500
B600	pine bark	none	600
B700	pine bark	none	700
R700	forest residue	none	700
C700	corncobs	none	700
LB500	pine bark	acid leaching	500
LB600	pine bark	acid leaching	600
LB700	pine bark	acid leaching	700
LR700	forest residue	acid leaching	700
LC700	corncobs	acid leaching	700

The mass yield of char, *y*_c_, was calculated from the experimental data as
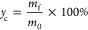
1where, *m*_0_ is the initial mass of the sample and *m*_f_ is the final mass of the sample.

### 2.2 Sample Characterization

#### 2.2.1 Proximate Analysis

The volatile matter content of the char samples was measured based on TGA^46^ using TGA8000, PerkinElmer. This measurement was carried out under an N_2_ atmosphere by first heating the sample, 2–3 mg, from 30 to 105 °C and held for 10 min to remove moisture in the sample. Then, the temperature was increased to 900 °C at a heating rate of 25 °C min^–1^ and held at this temperature for 10 min.^46^ The ash content was measured according to DIN 51719 using the macro-TG reactor, which was presented in the previous section. The sample of approximately 1 g was heated from 25 to 550 °C under an air atmosphere with a heating rate of 10 °C min^–1^. The sample was kept at the final temperature until there was no mass change. The fixed carbon of the sample was calculated by difference.

#### 2.2.2 Elemental Composition

The organic elemental analysis was carried out using a EA3000, CHNS-O elemental analyzer from Eurovector Srl. The determination of CHN was measured according to DIN 51732. The oxygen content was measured separately in the same analyzer using silver capsules injected into the reactor held at 1070 °C, which contained pure helium and was packed with nickel-plated carbon. The oxygen content was then determined by the content of CO in the gas products by means of gas chromatography using a thermal conductivity detector.

The inorganic elemental analysis was carried out using inductively coupled plasma optical emission spectrometry (ICP-OES) after microwave-assisted pressurized acid digestion. A Multiwave PRO microwave system (Anton Paar, Graz, Austria) was used for digestion. Each material was analyzed by digesting around 20 mg of the char samples with 7 mL of concentrated nitric acid, 0.2 mL of hydrofluoric acid, 0.2 mL of hydrochloric acid, and 0.2 mL of HClO_4_ (Carl Roth, Karlsruhe, Germany). The sample was heated to 195 °C within 15 min with the application of 1500 W of power, followed by a dwell time of 25 min at 195 °C. The digested samples were further diluted to 14 mL with deionized water. The ICP-OES system was an Arcos SOP by SPECTRO (Kleve, Germany). Sample blanks and spikes were included in all preparation procedures. The certified reference material NCS DC 73348 “Bush Branches and Leaves” (China National Analysis Centre for Iron and Steel, Beijing) was used for quality control.

#### 2.2.3 Carbon Structure

Raman spectroscopy was applied to analyze the molecular structure and morphology of char samples. Raman spectra were collected using an inverted microscope (IX71, Olympus, Japan) coupled to a spectrometer (Shamrock 303i, Andor Technology, UK). A DPSS 532 nm was used as an excitation laser (Altechna, Azpect Photonics AB, Sweden). The laser was operated at 6 mW. Spectra were collected from five different spots for each sample with 120 s of exposure time. All spectra were analyzed between 1100 and 1800 cm^–1^. Cosmic ray spikes were removed using the method provided by Schulze and Turner,^47^ and the spectra were smoothed using a Savitzky and Golay filter.^48^ The fluorescence signal was eliminated by baseline subtraction, according to Cao et al.^49^ All spectra were normalized using a maximum intensity of around 1590 cm^–1^ as the reference. The Raman spectra of amorphous carbons are usually deconvoluted into several bands to improve fitting. A variety of deconvolution methods have been proposed in the literature.^50−55^ However, the number of band assignments and band shapes can easily influence the outcome of the result, leading to overprediction. Here, only three Gaussian bands were assigned to the relevant Raman bands that appeared in the spectra, that is, the D band at 1350 cm^–1^, G band at 1590 cm^–1^, and V band at a valley around 1450 cm^–1^. The band assignment was performed by implementing the peak fit function^56^ in MATLAB.

X-ray diffraction (XRD) patterns of char samples were collected by using PANalytical Empyrean diffractometer with a copper tube. A Si low-background sample holder was used. The diffraction angle, 2θ, was between 10 and 90°, with 0.0066° of step size. The chosen step size enables the detection of graphite, carbon nanocrystals, and amorphous carbon. Inorganic compounds can also be visualized as sharp spikes. Background subtraction and signal smoothing were done according to Cao et al.^49^ and using the Savitzky–Golay filter,^48^ respectively. The XRD diffractograms were fitted with two Gaussian bands at 2θ of 24 and 44°, which represent (002) peak and (100) peak, respectively, by using the peakfit function^56^ in MATLAB. The full width at half-maximum (fwhm) of (002) and (100) peaks was extracted for analysis. The (002) peak refers to the reflections from stacked graphene layers, while the (100) peak originated from reflection from aromatic ring clusters within graphene layers.^57−59^ Quantification of carbon structure terms, that is, graphene stack height (*L*_c_) and width (*L*_a_), can be determined from the Scherrer equation,^60,61^ read as
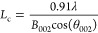
2
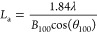
3where, λ is the XRD wavelength (1.542 Å), while *B*_002_ and *B*_100_ are fwhm of (002) and (100) peaks, respectively. The terms θ_002_ and θ_100_ are the reflection angles of (002) peak and (100) peak, respectively.

#### 2.2.4 Surface Morphology

The surface morphology and microstructure of the char samples were examined by scanning electron microscopy (SEM) using a Zeiss Ultra 55 VP equipped with energy-dispersive X-ray spectrometry (EDX), Bruker. The samples were spread and stuck on a carbon tape, which is placed on a sample holder. The secondary electron mode was used to examine the microstructure and morphology of char particles. In addition, the SEM analysis was operated in a backscattered electron mode for illustrating a distribution of selected inorganic elements in a scanned area. EDX semiquantitative analyses were carried out for interesting spots and areas to get more detailed microchemistry information.

The specific surface area and specific pore volume of the char samples were determined using an N_2_ physisorption method. It should be noted that the accuracy of the surface area measured by N_2_ physisorption decreased for pore sizes below 1.47 nm.^62^ The measurement was carried out using a Micromeritics ASAP 2000 analyzer. Prior to the measurements, around 200–400 mg of the sample was degassed overnight at a temperature of 150 °C and low pressure at 133 Pa. Adsorption isotherms were obtained by immersing sample tubes in liquid nitrogen (−197 °C) to obtain isothermal conditions. Nitrogen gas was added to the samples with a small pressure increment, resulting in adsorption isotherms. The adsorption isotherms are provided in the Supporting Information, Figure S1. The specific surface area was calculated by using the Brunauer–Emmett–Teller method.^63^ To increase the accuracy of the results, the adsorption isotherm between the lowest relative pressure and the highest relative pressure that did not give a negative C-value, that is, the energy of monolayer adsorption, was used in the calculation. The specific pore volume was defined as the volume of the adsorbate at the highest relative pressure, >0.99.^64^

### 2.3 Measurement of Gasification Rate

The intrinsic reaction rate of char gasification under CO_2_ was measured by using TGA8000 coupled with a gas mixing device GMD8000 from PerkinElmer Inc. The char samples with particle size below 75 μm were used to minimize the effect of intraparticle diffusion. Around 0.5–1.5 mg of the sample was loaded and spread at the bottom of an alumina crucible (diameter of 7 mm and height of 2 mm) as a thin layer to minimize the effect of interparticle diffusion. Reaction gas was fed in the vertical direction down to the crucible, and the total flow rate was kept constant at 100 mL min^–1^ in the standard state (25 °C and 10^5^ Pa). The sample was heated from 35 to 800 °C, at a heating rate of 10 °C min^–1^ under N_2_ (purity ≥ 99.996%). Once the target temperature was reached, the gas composition was switched to 20% (vol.) of CO_2_ (purity ≥ 99.99%) in N_2_. The sample was held at the isothermal condition for 5 h. After eliminating the heating part from the TG curves, Figure S2 in the Supporting Information depicts the results of the char samples produced from five repetitions.

The TG curves show deviation within the repetition due to the heterogeneous properties of the feedstocks. The results contain mass losses due to the overlapping reaction between devolatilization and gasification. Therefore, additional experiments were carried out under pure N_2_ at the same temperature programs. The devolatilization rates were evaluated from the experimental data by using the first-order reaction equation. The detailed evaluation and the devolatilization curves are provided in the Supporting Information, Figure S3. The devolatilization rate was eliminated from the overall results, and only gasification data is acquired by
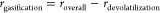
4where, *r* is the reaction rate in mass basis (g s^–1^). An example of the TG results after removing devolatilization is shown in Figure S4 in the Supporting Information. Then, the conversion of biomass char (*X*) during gasification was calculated by
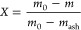
5where, *m*_0_ is the initial mass at the beginning of gasification, *m*_ash_ is the mass of ash obtained from the final mass of the experiment, and *m* is the mass monitored at a given time during gasification. The average value was determined from five repetitions, which is used to present the result.

## 3 Results and Discussion

### 3.1 Char Yields

**Figure 2:**
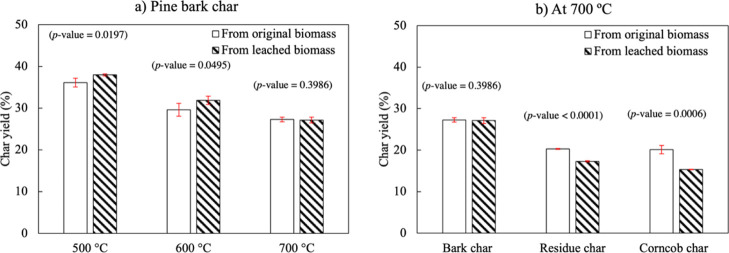
Mass yields of char produced from (a) pine bark at different pyrolysis temperatures and (b) different biomasses at 700 °C.

Figure 2 shows char yields on mass basis obtained from different pyrolysis temperatures and biomasses. As shown in Figure 2a, char yield decreased with increasing pyrolysis temperature, which is consistent with the literature.^10^ In the comparison among different biomasses, Figure 2b, pine bark gave maximum char yield of 27.3%, while forest residue and corncobs gave lower yields of 20.3 and 20.1%, respectively. As shown in Table 1, pine bark contains much higher lignin amount than the other biomasses, resulting in higher char yield, which is generally mentioned in the literature.^10,65,66^ It is noteworthy to mention that the mass yields of char produced from these biomasses are higher than those of stem woods reported in our previous study^10^ (i.e., spruce = 19.3% and birch = 16.9% at pyrolysis temperature of 700 °C), which corresponds with the amount of the lignin and ash content in raw biomasses. The yields of char produced from original pine bark and leached pine bark did not show a significant difference (a *p*-value lower than 0.05 implies that the difference is statistically significant with 96% confidence). In contrast, acid leaching significantly decreased the yields of forest residue char and corncob char to 17.3 and 15.3%, respectively. The previous studies often observed the increase in char yield with the presence of AAEMs.^67,68^ It can explain the results as the majority of AAEMs in forest residue and corncobs is K, which was nearly completely removed upon acid leaching. Meanwhile, a considerable amount of Ca remained in leached pine bark and kept potential to increase the char yield (see Table 2).

### 3.2 Textural Structure of Biomass Char

**Figure 3:**
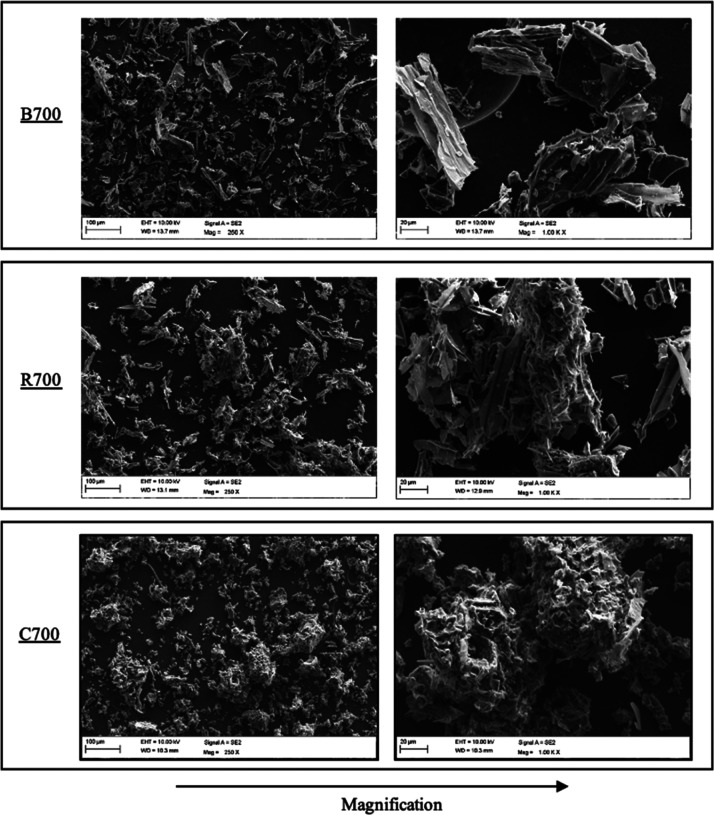
SEM images of different biomass chars produced at 700 °C (B: pine bark, R: forest residue, and C: corncobs).

Figure 3 displays SEM images of char produced from different biomasses at pyrolysis temperature of 700 °C, that is, B700, R700, and C700. The surface morphology of pine bark char and forest residue char showed the same typical fiber structure of forestry biomass as reported in the literature.^15,69^ After the fragmentation of particles, they tend to have a thin flake-like structure, which was original cell walls. Meanwhile, corncob char showed a distinct sphere-like shape. The higher magnification images show that corncob char contains more and larger pores on the particle surface compared with pine bark and forest residue char, which have a smooth surface.

The specific surface area and pore volume of the char samples are shown in Table 4. Char produced from higher pyrolysis temperatures resulted in a higher specific surface area. This is because more volatile matters are released from char at higher pyrolysis temperatures, leaving char with more pores. Among the different biomass origins, pine bark chars have the highest surface area, followed by forest residue and corncob char. Char samples produced from leached biomasses have even higher specific surface areas than those obtained from original biomasses. This result implies that acetic acid extracted inorganic compounds from the biomasses matrix, which left more open pores in the biomasses and their char, as also reported in the literature.^70^

**Table 4:** Specific Surface Area and Pore Volumes of the Char Samples

sample	specific surface area (m^2^/g)	specific pore volume (cm^3^/g)
B500	1.8	4.8
LB500	37.4	16.9
B600	227.4	70.1
LB600	340.6	105.0
B700	387.7	111.4
LB700	446.7	121.7
R700	294.8	86.6
LR700	340.5	96.6
C700	34.1	21.1
LC700	254.7	83.9

### 3.3 Chemical Composition and Carbon Structure

Table 5 summarizes the proximate analysis of biomass char produced in this study. The char samples contain volatile matters in the range between 9.3 and 28.9%. The volatile matter content generally decreased when pyrolysis temperature increased due to thermal degradation of the biomass. Char produced from different types of original biomasses did not give a significant difference in the volatile matter content (*p*-values > 0.0777). The ash content of char in this study is varying from 0.9 to 7.0%. In contrast to volatile matters, the ash content increased when pyrolysis temperature increased because the major inorganic compounds remained in the biomass char at pyrolysis temperatures below 900 °C.^25,37−40,71^ Therefore, once volatile matters were released from biomass at higher pyrolysis temperatures, the char samples were left with ash and carbon. Acid leaching significantly decreased the ash content of the char, except for those produced from forest residue. This exception occurred due to the extremely heterogeneous raw forest residue, as can be observed from the large standard deviation. Similar to the ash content, the fixed carbon content increased with pyrolysis temperature. The fixed carbon content of char produced from the different biomasses did not show a significant difference. Also, acid leaching does not seem to have a significant effect on a fixed carbon content. Biomass char produced in this study contain a fixed carbon content between 67.3 and 87.5%.

**Table 5:** Proximate Analysis of the Char Samples (% of Dried Mass Basis)

sample label	VM content	ash content	fixed carbon content
B500	28.9 (±0.4)	3.8 (±0.3)	67.3 (±0.4)
LB500	27.8 (±0.9)	2.6 (±0.2)	69.7 (±0.9)
B600	19.0 (±1.1)	4.8 (±0.5)	76.3 (±1.2)
LB600	18.4 (±1.8)	3.1 (±0.2)	78.6 (±1.8)
B700	12.6 (±0.7)	5.6 (±0.7)	81.8 (±1.0)
LB700	12.4 (±0.4)	3.7 (±0.1)	83.9 (±0.4)
R700	12.2 (±0.9)	3.5 (±0.7)	84.3 (±1.1)
LR700	9.3 (±0.9)	7.0 (±4.6)	83.8 (±4.7)
C700	11.8 (±0.9)	5.7 (±0.3)	82.6 (±0.9)
LC700	11.6 (±1.0)	0.9 (±0.4)	87.5 (±1.1)

**Figure 4:**
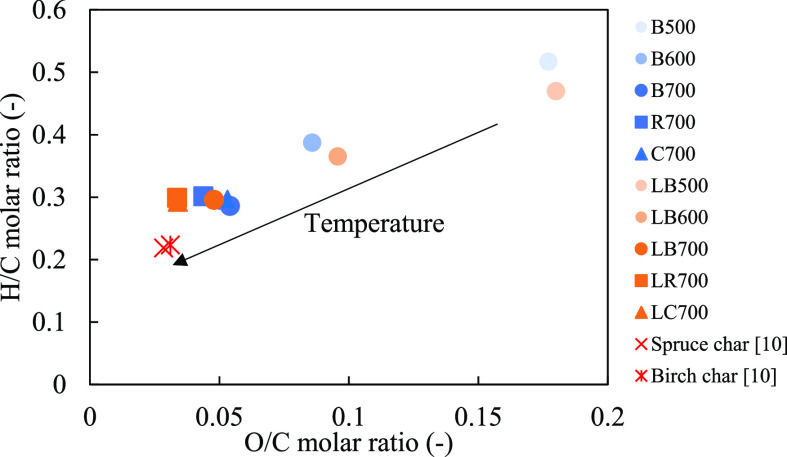
Influence of pyrolysis temperature on the elemental composition of biomass char, shown in a Van Krevelen diagram.

According to the ultimate analysis provided in Table S1 in the Supporting Information and Figure 4 shows the Van Krevelen diagram of the char samples. The char produced in this study have H/C ratios ranging between 0.29 and 0.52, while O/C ratios are between 0.03 and 0.18. Pyrolysis temperature is the main influencing factor on H/C and O/C ratios, while the difference in biomass type and acid leaching gave low effects. The H/C and O/C ratios of char samples decreased when pyrolysis temperature increased due to thermal degradation of hydroxy and oxygenated groups in the biomasses. The figure also shows that the H/C ratios of char produced in this study are higher than those of stem wood char.^10^ However, the O/C ratio of char produced from acid leached forest residue and corncobs are at a similar level as char produced from stem woods.^10^

Table 6 summarizes the major inorganic elements and total inorganic elements determined by the ICP-OES method. The total inorganic elements of char varied between 7618 and 34 009 mg kg^–1^, which is higher than those of stem wood char (i.e., 5034–6040 mg kg^–1^) reported in the previous study.^10^ Ca and K are the most dominant inorganic elements in char samples produced in this study, followed by various amounts of Mg, Si, P, and Na. The content of S is very low and may, therefore, not have significant influences on the gasification rate. In general, the contents of these inorganic elements increased when pyrolysis temperature increased. In addition, K and Mg are significantly lower in the char produced from leached biomasses, while Ca remains high. The amount of P is lower in the char produced from leached biomass except for forest residue char, which is due to the heterogeneous raw biomass. It should be noted that the Na content increased after acid leaching, which may be due to reactions between the acid and soda–lime glassware. However, Mg and Na contents are much lower than Ca and K, and they should not have a significant effect on the gasification reactivity.

**Table 6:** Major Inorganic Compositions and Total Inorganic Elements of Char (mg kg^–1^)

sample label	Ca	K	Mg	Na	Si	P	S	total
B500	10100	4430	1200	90	664	877	143	19926
LB500	10300	541	398	222	302	386	140	13550
B600	12300	5450	1450	124	983	1080	155	24677
LB600	12400	617	495	262	419	475	133	16338
B700	14760	6460	1742	150	1234	1316	181	29080
LB700	13100	734	534	285	399	500	130	17222
R700	8820	6380	1710	859	607	20	147	20905
LR700	4895	3210	783	1525	15850	301	78	34009
C700	694	25700	1670	49	1880	1490	207	32533
LC700	1180	2750	421	206	1980	469	128	7618

**Figure 5:**
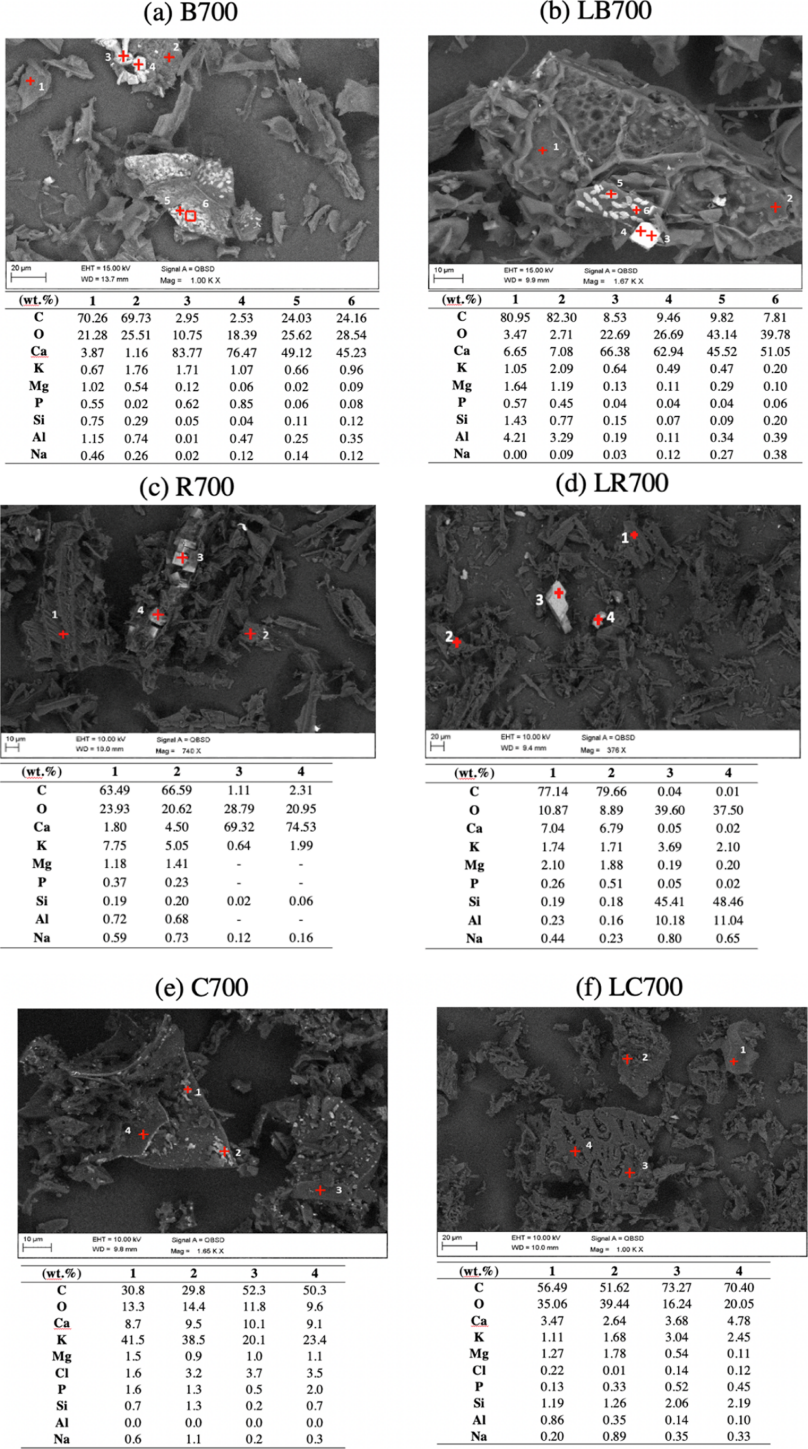
SEM–EDX results of (a) B700, (b) LB700, (c) R700, (d) LR700, (e) C700, and (f) LC700. (B: pine bark, R: forest residue, C: corncobs, and L: leached).

Figure 5 shows distribution of ash-forming elements on char samples measured by SEM–EDX. For pine bark and forest residue char (Figure 5a–d), Ca is the most dominant inorganic element, which is randomly dispersed through the char particles. In addition, large Ca oxalate crystals were observed in the figures. This implies that pyrolysis temperatures used in this study did not completely break Ca oxalate into small CaO particles, and there is no uniform dispersion of CaO. This result agrees with the observation from our previous study^34^ which showed that CaO dispersion was not changed by thermal treatment at below 1600 °C. The figures also show that Ca was not removed from these biomasses by acetic acid leaching. It should be noted that Si randomly contaminated forest residue char. For char samples produced from corncobs (Figure 5e), K is the most dominant inorganic element, which is uniformly dispersed through the char particles. K was barely observed in leached corncob char (Figure 5f), which indicates that acetic acid effectively removed K from these biomasses.

The same observation is also detected by XRD (Figure S5). Acid leaching did not significantly remove Ca from pine bark char particles, which agreed with the result measured from ICP-OES (Table 6). A high Ca content was also observed in forest residue char, but acid leaching seems to remove some Ca from the char particles. This may be due to different Ca compounds or different bondings between Ca compounds and a carbon matrix and between pine bark and forest residue. On the one hand, the Ca content in pine bark originate from the biological development of plants. On the other hand, Ca in forest residue char is most likely due to contamination during material handling of raw forest residue. This nonhomogeneous nature of forest residue can also be observed by Si content, which may be contaminated from sand and soil. Corncob char showed a high content of K, while char from leached corncobs showed very low K content, which agree with the results obtained from ICP-OES. It is interesting to notice that corncob char contains a low amount of Cl, which could not be detected in the other biomass char.

The (002) and (100) peaks in XRD diffractograms were used to interpret the carbon structure of biomass char. Table 7 shows the values of crystallite width, *L*_a_, and crystallite stack height, *L*_c_ of char, all together with the results from Raman spectroscopy. In general, the crystallite width slightly increased when pyrolysis temperature increased. However, the results of crystallite stack height do not show any trend even for the char produced from different temperatures. The latter occurred because the graphene layers in amorphous carbons randomly align in the vertical direction, unlike the well-stacked structure as in graphitized carbons.

**Table 7:** Summary of the Carbon Structure Including *L*_a_, *L*_c_, *I*_D_/*I*_G_, and fwhm_D_ of Char

sample label	*L*_a_ (nm)	*L*_c_ (nm)	ID/IG (−)	fwhm_D_ (cm^–1^)
B500	2.61	1.79	0.53 (±0.02)	219 (±6.78)
LB500	2.54	1.71	0.46 (±0.01)	217 (±11.84)
B600	2.75	1.70	0.55 (±0.02)	213 (±10.01)
LB600	2.64	1.73	0.52 (±0.02)	214 (±5.76)
B700	2.77	1.75	0.62 (±0.03)	199 (±23.83)
LB700	2.76	1.74	0.60 (±0.02)	206 (±3.92)
R700	2.83	1.81	0.62 (±0.03)	214 (±4.96)
LR700	2.76	1.82	0.59 (±0.01)	209 (±3.84)
C700	2.68	1.75	0.64 (±0.03)	206 (±9.67)
LC700	2.78	1.84	0.61 (±0.03)	211 (±4.62)

The aromatic carbon structure was also analyzed from Raman spectroscopy. An example of a Raman spectrum measured from char produced in this study is provided in Figure S6 in the Supporting Information. The spectra showed two overlapping peaks with maximum intensities located at 1350 and 1590 cm^–1^, referring to the D band and G band, respectively. It is widely reported in the literature that the G band reflects the motion of carbon sp^2^ atoms, including rings and chains of amorphous carbons.^72−75^ The D band represents the amount of large aromatic clusters, that is, more than six fused rings.^75^ Therefore, the intensity ratio of the raw spectra, *I*_D_/*I*_G_, has been widely applied to indicate the amount of large aromatic clusters in amorphous carbons. High values of the *I*_D_/*I*_G_ ratio refer to the high amount of large aromatic clusters in carbons. As shown in Table 7, the *I*_D_/*I*_G_ ratio clearly distinguishes the amount of large aromatic clusters of char produced from different pyrolysis temperatures. It shows that the *I*_D_/*I*_G_ ratio increased when the pyrolysis temperature increased, which is supporting the result of crystallite width obtained from XRD. This result indicates an increase in structural order and larger aromatic ring clusters at higher pyrolysis temperatures. However, our previous study^25^ found that the *I*_D_/*I*_G_ ratio did not show significant differences among biomass char produced at the same pyrolysis temperature, but fwhm of the D band (fwhm_D_) could distinguish the carbon structure of the char produced from the same pyrolysis temperatures. The lower the value of fwhm_D_, the higher the order of the aromatic carbon clusters . Therefore, the fwhm_D_ band measured from the char produced in this study was determined, and the results are also provided in Table 7. Although the fwhm_D_ clearly distinguishes the carbon structure of the char produced from different pyrolysis temperatures, it does not show a clear trend among char produced from different biomasses at 700 °C. This result indicates that the aromatic carbon structures of char samples produced in this study are not distinctly different from each other, implying similar resistance against chemical reactions at the molecular scale.

### 3.4 Gasification Rates of Pine Bark Char Produced at Different Pyrolysis Temperatures

**Figure 6:**
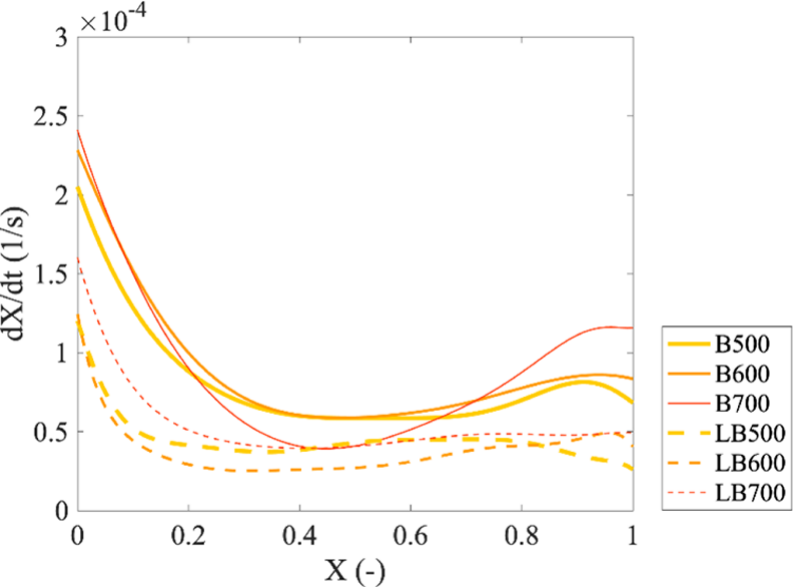
Conversion rates of char produced at different pyrolysis temperatures.

Figure 6 displays the gasification rate as a function of the conversion of pine bark char produced from different pyrolysis temperatures. For the char produced from the original pine bark (i.e., B500, B600, and B700), the conversion rate progress changed along with conversion, which could be divided into three sections. The first section took place at the initial conversion rate, that is, *X* = 0. Char produced from higher pyrolysis temperatures resulted in higher initial conversion rates. As the reaction continued, the conversion rate of char sharply decreased attributed to the annealing effect of carbon because the char samples were produced at pyrolysis temperatures lower than the gasification temperature.^76−79^ An additional experiment was conducted to verify the annealing effect, and the detail is provided in the Supporting Information, Figure S7. At conversion higher than 0.5, the conversion rate increased with conversion. In fact, the conversion rate of B700 increased with a magnitude higher than that of B500 and B600. For the char produced from leached pine bark (i.e., LB500, LB600, and LB700), the conversion rates began at much lower values than those of char produced from the original pine bark. Then, the rate decreased due to the annealing effect and remained constant until completed conversion.

As presented in the previous sections, specific surface area, fixed carbon content, and carbon content increased with pyrolysis temperatures. In addition, larger aromatic clusters and more ordered carbon structures were present in char produced at high pyrolysis temperatures, which should result in low reactivity. Nevertheless, the initial gasification rate of char showed an opposite correlation with the carbon structure. This result implies that the gasification rates of these pine bark chars were not mainly controlled by carbon structure and degree of aromaticity, but by a combination effect with other properties.

**Figure 7:**
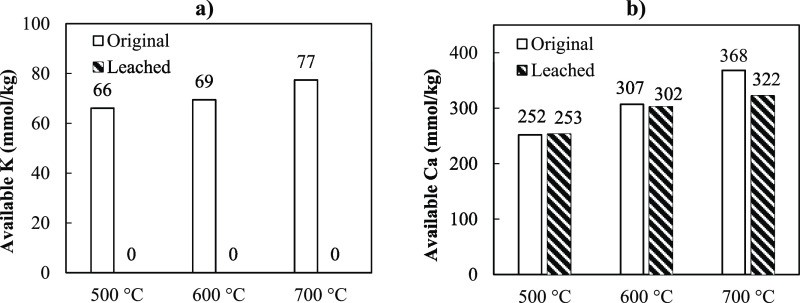
Available amount of (a) K and (b) Ca in pine bark char produced from pyrolysis at different temperatures.

As explained earlier, K and Ca play catalytic roles to promote char conversion during the gasification process. Whereas, Si may decrease the availability of the K and Ca by forming silicates, which reduce catalytic effects of the two elements. According to the literature,^33^ Si has higher affinity to react with K and tend to form silicate before Ca. By assuming that all Si in char formed simple silicates with K and Ca, such as K_2_SiO_3_ and CaSiO_3_, respectively, the minimum amount of K and Ca available to catalyze char gasification can be estimated by
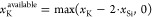
6
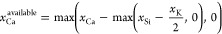
7where, *x*_*i*_ represents the molar content of element *i* in char (mmol kg^–1^). The estimations in eqs 6 and 7 are constructed for K and Ca dominated fuels, and it is based on the assumption that release and reactions with other interacting inorganic elements can be neglected. It should be mentioned that P, Cl, and S contents in char studied in the present study were low; hence, they were assumed not to interfere with the assumptions, even though reactions always occur. Figure 7 depicts the estimated available amounts of K and Ca in pine bark char. It can be observed that the amount of active K and Ca in the char increased when pyrolysis temperature increased, implying the low release of these elements at the pyrolysis conditions applied in this study. Hence, pine bark char produced at higher temperatures exhibit higher reaction rates.

The figure also displays that char produced from leached pine bark contained no active K (Figure 7a), while active Ca is slightly lower than in the char produced from the original pine bark (Figure 7b). This observation occurred because leached pine bark char contains very low K, and Ca is the only catalytic element available in leached pine bark char. This result revealed that the amount of active K significantly affects the conversion rate of char samples, and the higher content of active K also refers to higher conversion rates. Although leached pine bark char contains the same amount of active Ca as original pine bark char, the reaction rates are lower than those of char from original pine bark. This result shows that the Ca content did not affect the gasification rate of pine bark char produced in this study. According to the previous study,^34^ CaO dispersion on the char surface did not change at temperature below 1600 °C, corresponding to the low catalytic activity of Ca. This implies that the catalytic activity of Ca does not only depends on its content in char, but mobility and vaporization of Ca is the important factor that defines the catalytic effect of Ca during gasification reaction. SEM–EDX analysis (Figure 5a,b) revealed that there is less intensive CaO dispersion on particles of pine bark char. Hence, at the low temperatures applied in this study, Ca does not actively catalyze the gasification of the char samples due to its low mobility and dispersion.

Without major effects from active K, the gasification reactivity of leached pine bark char should be mainly influenced by its morphology and carbon structure. However, the correlation between temperature and char reactivity in leached pine bark char is unclear. Leached pine bark char produced at a high temperature contain large aromatic clusters, but it also has a high surface area, which can lead to high reactivity. Therefore, counteracting effects between the aromatic carbon structure and specific surface area may cause unclear correlation for leached pine bark char.

### 3.5 Gasification Rates of Char Produced from Different Biomasses

**Figure 8:**
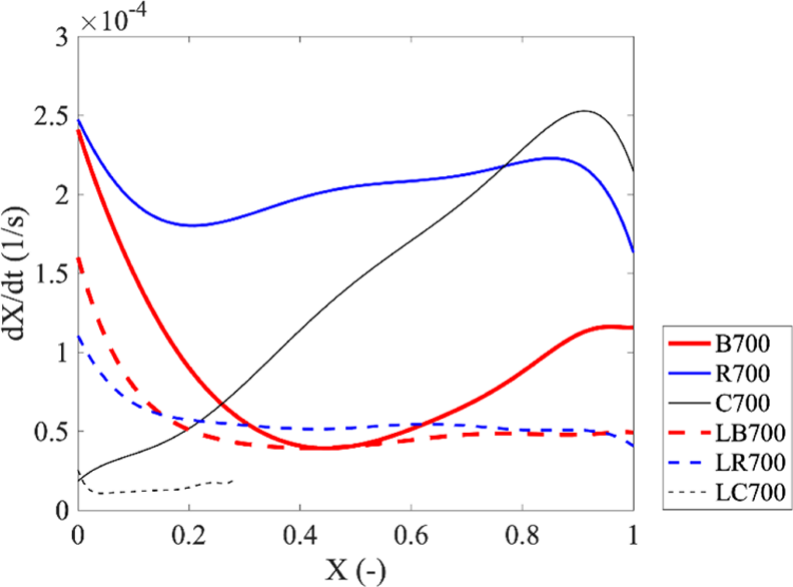
Conversion rates of char produced from different biomasses.

Figure 8 depicts conversion rates of char produced from different types of biomasses at pyrolysis temperature of 700 °C. In the char produced from different original biomasses, their conversion rates progressed very differently. The initial conversion rates of B700 and R700 are at the same level. In both B700 and R700, the rate firstly decreased when conversion increased due to the annealing effect, and it then increased at higher conversions. It was observed that the conversion rate of R700 stopped decreasing at ca. *X* = 0.2, which is earlier than B700 at ca. *X* = 0.4. However, the rate of B700 continued to increase until the conversion was completed, while the rate of R700 sharply decreased at *X* > 0.9. In contrast to the forestry biomass char, the initial conversion rate of corncob char, that is, C700, was ca. 10 times lower than those of B700 and R700. Also, the rate of C700 continuously increased with the conversion and reached a maximum at *X* = 0.9 before it decreased, which agrees with results reported in previous studies.^80,81^ It should be noted that the maximum rate of C700 is higher than the maximum rates of B700 and R700. For the char produced from leached biomasses, the conversion rates measured from them show the same shape. The rate decreased when conversion increased due to the annealing effect, and it stays a plateau or slightly decreased at higher conversions. The initial rates of LB700 and LR700 are ca. 1.6 and 2.5 times lower than those of B700 and R700, respectively. The initial rate of LC700 does not show much difference from that of C700. However, it is noteworthy to mention that the final conversion (after 5 h) of LC700 was lower than 0.3, implying significantly lower gasification reactivity in comparison with other char samples.

As described in Section 3.2, the carbon structure of char produced at the same pyrolysis temperature did not show a distinct difference. This means that the order of carbon structure did not play a major role in the conversion rate of these char samples. Therefore, the morphology and inorganic composition of these chars are the major factors influencing the gasification rate.

**Figure 9:**
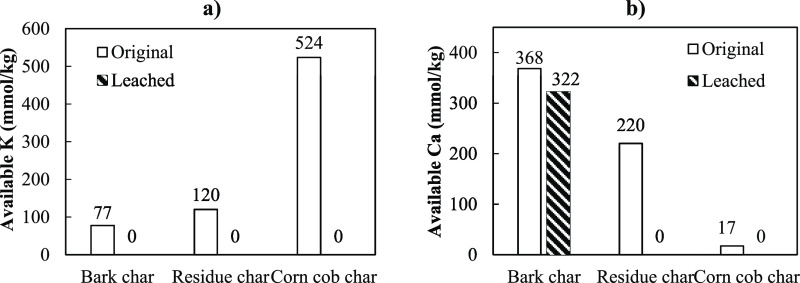
Available amount of (a) K and (b) Ca in char produced from different types of biomasses at a pyrolysis temperature of 700 °C.

Figure 9 depicts the contents of active K and Ca in the char samples, as calculated from eqs 6 and 7. As shown in Figure 9a, the active K content in C700 is around 4–5 times higher than those of B700 and R700. This high active K content explains the higher conversion rate measured from C700. In addition, the conversion rate started to increase at earlier conversion for the char sample that had higher active K content. In the chars produced from leached biomasses, the active K content is barely existing. Therefore, the reaction rates of these chars are much lower than the chars from the original biomass. In Figure 9b, no active Ca is available in LR700 and LC700 due to their low Ca and high Si contents. However, the amount of active Ca in LB700 is as high as that of B700. The result is verifying that Ca was not the cause of the rate acceleration during gasification. This occurs because Ca in the char sample was not mobilized in the char because the gasification temperature is low, that is, 800 °C.

In leached char, neither the carbon structure nor the active K content differed among the samples, hence having no effect on their reaction rates. As observed from SEM images (Figure 3) and the specific surface area (Table 4), corncob char (LC700) has a much lower specific surface area than forestry biomass, that is, LB700 and LR700. This is the reason for much lower reactivity in LC700 compared to other leached biomasses.

## 4 Conclusions

This study examined the properties and gasification reactivity of biomass char produced from pine bark, forest residue, and corncobs. These biomass chars contain substantially higher Ca, K, and Si amounts compared with the char produced from stem wood. Leaching by using diluted acetic acid effectively reduced the total inorganic content, particularly K, Mg, and P, in the biomasses, resulting in a low inorganic content in the produced char. Pyrolysis temperature affects the elemental composition, porous structure, and aromatic carbon structure of the char samples. At the same pyrolysis temperature of 700 °C, inorganic composition and specific surface area varied with the type of biomass, while the other properties did not show a distinct difference.

In contrast to char produced from debarked woodchips, the gasification rate of pine bark char increased when pyrolysis temperature increased. This result occurred because inorganic content, particularly K, increased with pyrolysis temperature, resulting in higher catalytic activity, although the char samples contain larger aromatic clusters and higher specific surface area. This result implies that inorganic contents in high-ash biomasses play a more vital role than aromatic cluster size. For char produced from leached biomasses, gasification reactivity was controlled by counteracting effects between char surface area and aromatic carbon structure.

Char produced from different biomasses showed distinct gasification rates. According to the gasification temperature applied in this study, that is, 800 °C, it was found that K is the most influencing element on the reaction rate while Ca did not show significant effects. It is because K was able to mobilize through the char particles, while Ca was not vaporized and stayed inside the carbon matrix at this temperature. This outcome was confirmed by low gasification rates of leached char, which contain very low K and high Ca amounts. Hence, the observation from this study implies that pyrolysis and gasification temperatures are crucial factors determining char reactivity catalyzed by inorganic elements.

## Supporting Information Available

Adsorption isotherms, details of the experiment and calculation of the gasification rate, ultimate analysis of char samples, XRD diffractograms, deconvolution of Raman spectra, and annealing effect during gasification (PDF)
