# Supplementary material for: CO2 Gasification Reactivity of Char from High-Ash Biomass
Source: ACS Omega. 2021 Nov 29;6(49):34115–28. doi: 10.1021/acsomega.1c05728 (PMC8679002; doi:10.1021/acsomega.1c05728)
Supplement: Supplementary file 1 — ao1c05728_si_001.pdf [file ao1c05728_si_001.pdf]

SUPPORTING INFORMATION

CO_2 gasification reactivity of char from high-ash biomass

Aekjuthon Phounglamcheik*[a,b], Ricardo Vila[a], Norbert Kienzl[c], Liang Wang[d], Ali Hedayati[a], Markus Broström[e], Kerstin Ramser[f], Klas Engvall[g], Øyvind Skreiberg[d], Ryan Robinson[h], and Kentaro Umeki*[a]

[a]Luleå University of Technology, Division of Energy Science, SE-971 87, Luleå, Sweden
[b]Tampere University, Material Science and Environmental Engineering, FI-33720, Tampere, Finland
[c]BEST - Bioenergy and Sustainable Technologies GmbH, Inffeldgasse 21b, 8010, Graz, Austria
[d]SINTEF Energy Research, P.O. Box 4761 Torgarden, NO-7465, Trondheim, Norway
[e]Umeå University, Department of Applied Physics and Electronics, Thermochemical Energy Conversion Laboratory, SE-901 87 Umeå, Sweden
[f]*Luleå University of Technology, Division of Fluid and Experimental Mechanics, SE-971 87 Luleå, Sweden*
[g]*Department of Chemical Engineering, KTH Royal Institute of Technology, SE-100 44, Stockholm, Sweden*
[h]*Global Technology, Höganäs AB, SE-263 83, Höganäs, Sweden*

S1. Adsorption isotherms. Figure S1 depicts the adsorption isotherms of the char samples produced from different pyrolysis temperatures and different biomasses.

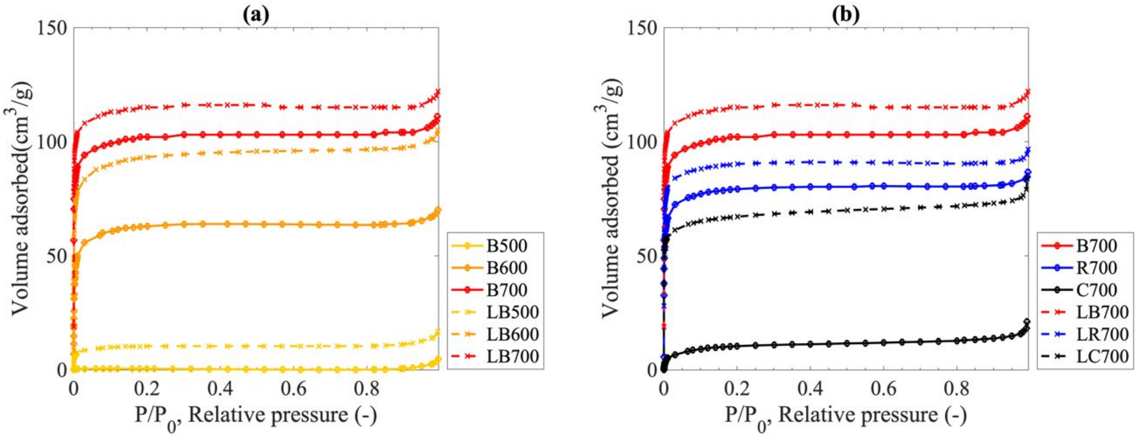

Figure S1. Adsorption isotherms of char samples from (a) different temperatures and (b) different biomasses.

S2. Detail of experiment and calculation of gasification rate

S2.1. The repeatability of the TG data. Figure S2 displays raw TG data obtained from 5 repetitions. It can be observed that the result contains great deviation due to the inhomogeneity of the feedstocks.

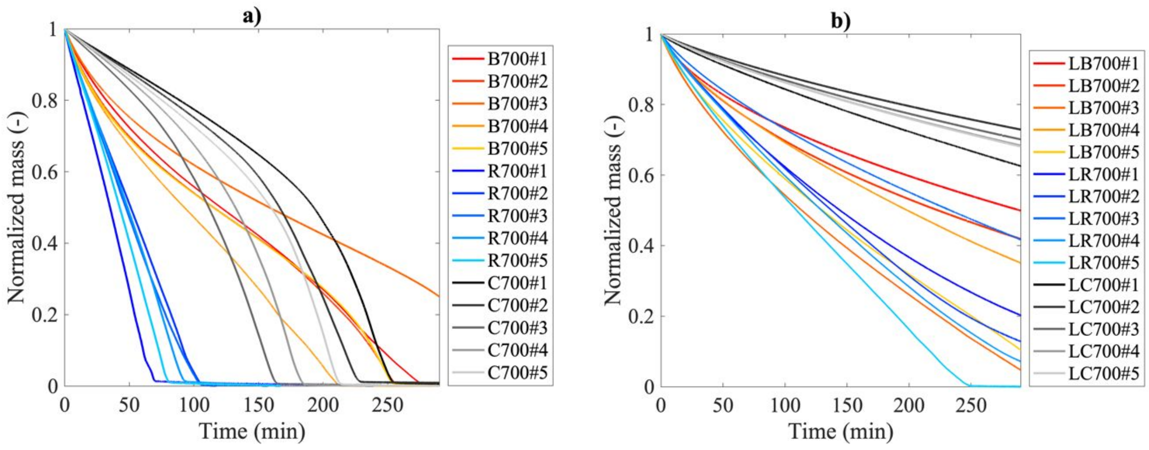

Figure S2. Raw TG data of char from (a) original biomasses and (b) leached biomasses.

S2.2. Elimination of devolatilization from TG data. According to the experiment measured under pure N_2, the devolatilization part is fitted by using the first-order kinetic model

$$\ln{(X_{dev})} = -\,k_{dev} \cdot t \qquad \text{(S1)}$$

where, X_{dev} is devolatilization conversion, $X_{dev} = (m - m_f)/(m_0 - m_f)$. The terms k_{dev} and t represent rate constant and time, respectively. Figure S3 shows normalized mass over time of devolatilization reaction evaluated by the model.

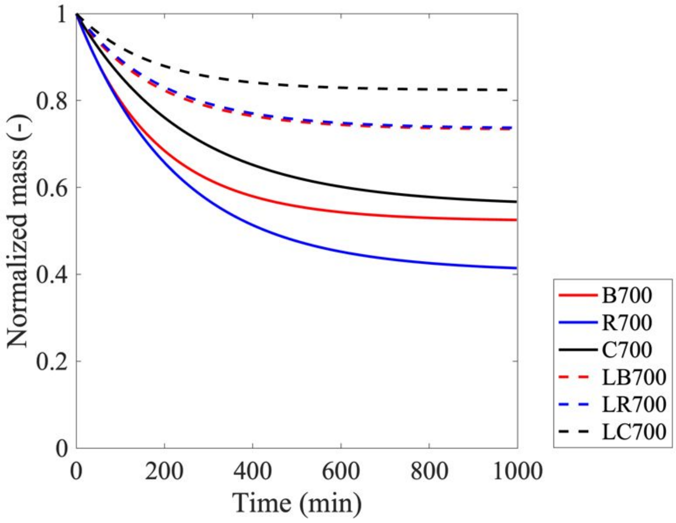

Figure S3. Mass loss rates of devolatilization.

The devolatilization part is then removed from the TG data of the measurement under CO_2 by using the following expressions

$$\frac{dm}{dt}_{gasification} = \frac{dm}{dt}_{overall} - \frac{dm}{dt}_{dev} \quad \text{(S2)}$$

Figure S4 shows an example of the thermogravimetric curve, which display a mass loss due to devolatilization and gasification.

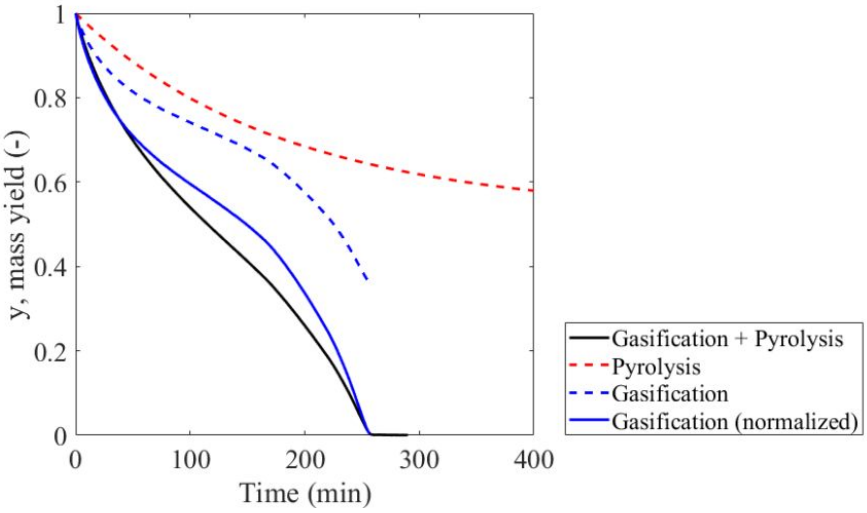

Figure S4. An example of the elimination of devolatilization for B700.

S3. Ultimate analysis of the char samples.

Table S1 summarizes the content of C, H, N, and O in the char samples. The values in brackets show standard deviations from 15 repetitions.

Table S1. Ultimate analysis of the char samples in % of dried mass basis

Sample label	C	H	N	O
B500	75.2 (±0.3)	3.2 (±0.1)	0.4 (±0.0)	17.8 (±0.1)
B600	82.7 (±0.4)	2.7 (±0.0)	0.4 (±0.0)	9.5 (±0.3)
B700	85.2 (±0.9)	2.0 (±0.0)	0.4 (±0.0)	6.2 (±0.6)
R700	88.0 (±0.3)	2.2 (±0.0)	0.4 (±0.0)	5.2 (±0.1)
C700	85.1 (±0.3)	2.1 (±0.0)	0.5 (±0.0)	6.0 (±0.1)
LB500	74.9 (±0.4)	2.9 (±0.1)	0.5 (±0.0)	18 (±0.6)
LB600	81.8 (±1.2)	2.5 (±0.1)	0.5 (±0.0)	10.4 (±1.1)
LB700	87.7 (±0.5)	2.2 (±0.0)	0.3 (±0.1)	5.6 (±0.1)
LR700	86.3 (±3.5)	2.2 (±0.1)	0.3 (±0.0)	3.9 (±0.1)
LC700	91.0 (±0.4)	2.2 (±0.1)	0.7 (±0.1)	4.1 (±0.1)

S4. XRD diffractograms.

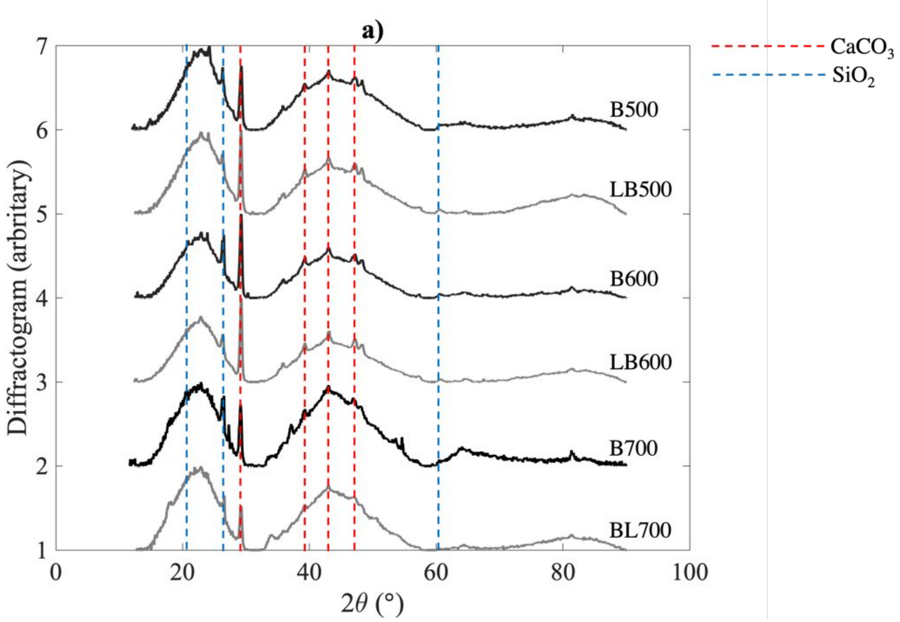

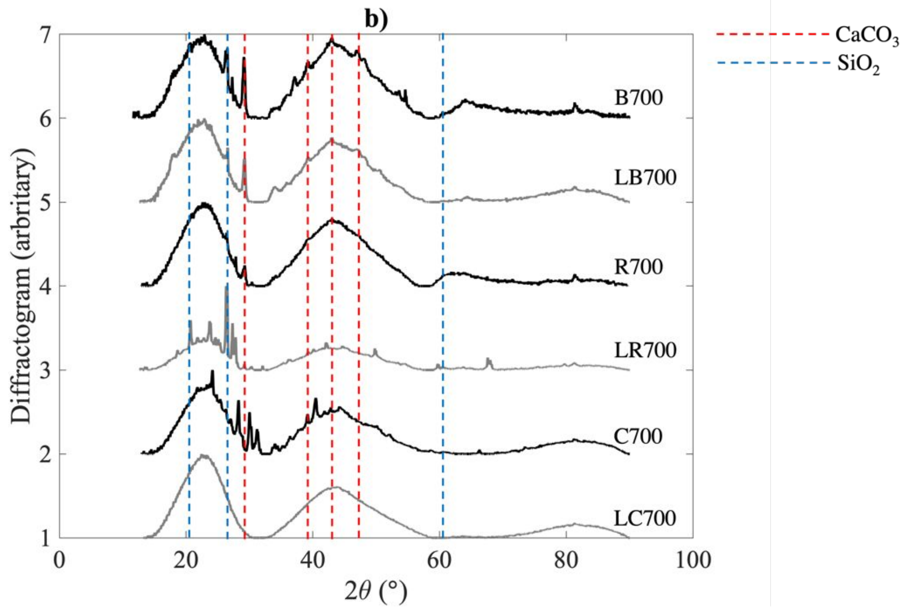

Figure S5. Diffractograms from XRD analysis of char produced from (a) different pyrolysis temperatures and (b) biomasses.

S6. Deconvolution of Raman spectra. Three Gaussian profiles were introduced in the spectra. The peak positions of the profiles were set at the position of G (1590 cm^{-1}), D (1350 cm^{-1}), and V (1450 cm^{-1}) of the spectra with 5 cm^{-1} of allowable tolerance. The peak fittings progressed from the initial width at 0 cm^{-1} until the R-squared of 0.995 was achieved. Once the results were obtained, the height of assigned bands at G and D positions were checked to make sure they are at around the intensities of the spectra. Figure S6 shows an example of Raman deconvolution of B700. The full width at the half maximum (FWHM) was obtained by measuring the width at the half-height of the Gaussian bands.

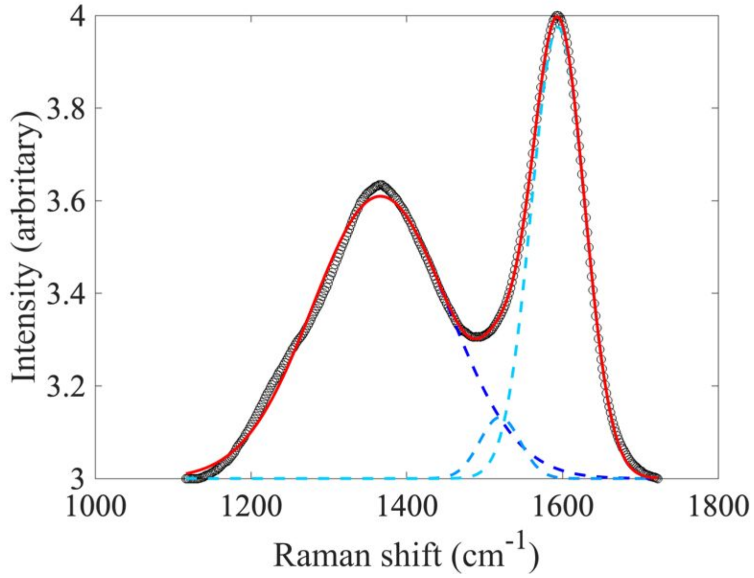

Figure S6. Deconvolution of the Raman spectrum from charcoal B700.

S7. Annealing effect during gasification. An additional experiment was carried out to describe the thermal annealing effect in the gasification results. This part has been done by using the same TG instrument, i.e., TGA8000 coupled with gas mixing device GMD8000 from PerkinElmer Inc. The heating part is duplicated from the experiment described in Section 2.3. Instead of switching the gas atmosphere from N_2 to 20% of CO_2 directly after the temperature reached 800 °C, the sample was held at the temperature under pure N_2 for 1 h before the gas switch to 20% of CO_2.

LB700, LR700, and LC700 were selected in this investigation due to their minimal effect of inorganic elements on the reaction rate. Figure S7 represent the gasification rate as a function of conversion. The figure was plotted for comparison between the rates obtained from the original procedure and this additional procedure. LR700 did not show the decrease in the rate caused by thermal annealing, while LB700 still show a small effect at low conversion. This could be attributed to the higher degree of thermal annealing that took place in LB700, similar to that shown in Figure 9. For LC700, thermal annealing did not appear and the gasification rate progress with conversion according to the pore development, which follows the random-pore model [S1].

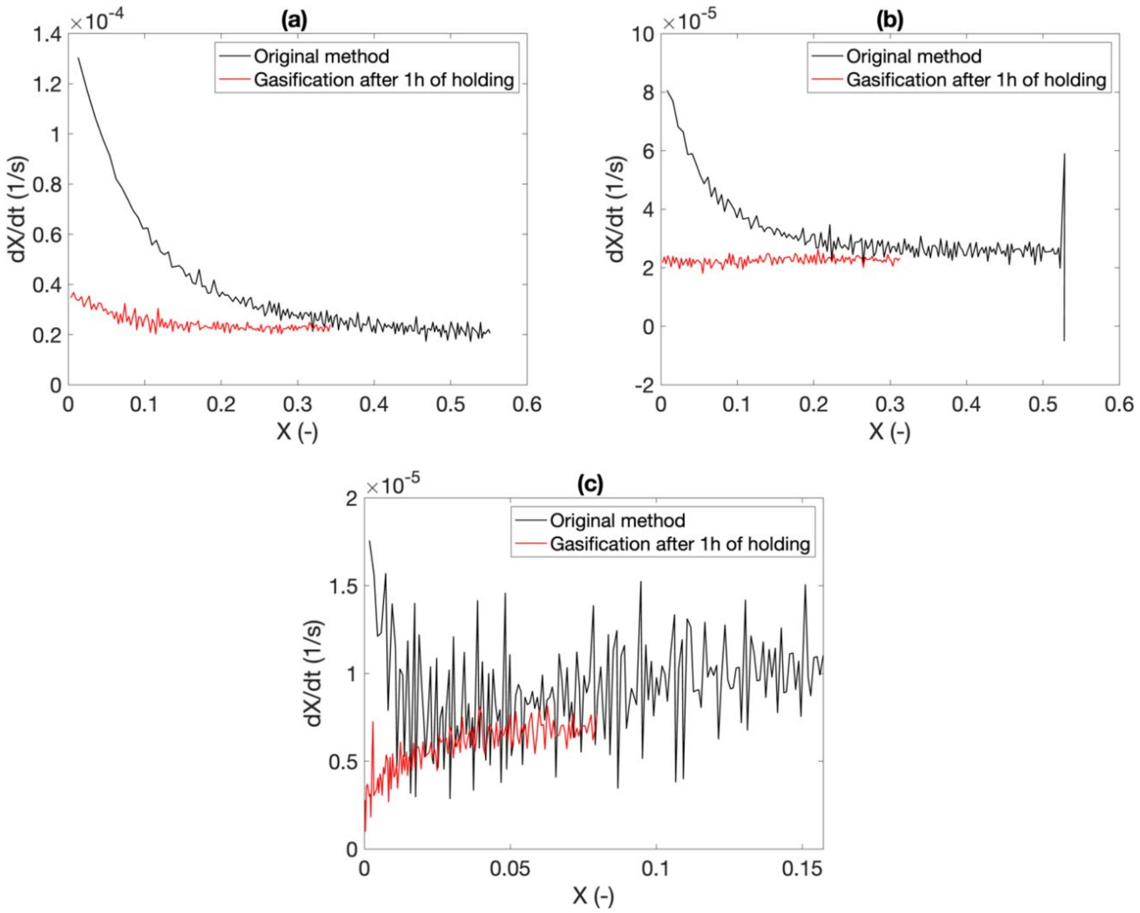

Figure S7. Effect of thermal annealing on gasification rates of (a) LB700, (b) LR700, and (c) LC700.

References

(S1) Bhatia, S. K.; Perlmutter, D. D. A Random Pore Model for Fluid-solid Reactions: I. Isothermal, Kinetic Control. *AIChE J.* **1980**, 26, 379-386.
